# Growing pains: exploring residents’ mental health in a rapidly growing municipality—a qualitative study

**DOI:** 10.3389/fpubh.2025.1659023

**Published:** 2025-11-10

**Authors:** Ann-Grete Dybvik Akre, Marie Dahlen Granrud

**Affiliations:** Department of Health and Nursing Sciences, Faculty of Health and Social Sciences, University of Inland Norway, Elverum, Norway

**Keywords:** mental health, municipality, qualitative content analysis, rapid growth, social capital, health services

## Abstract

**Background:**

Belonging to a community provides opportunities for engagement, social connectedness, and participation, which are important for mental health. Norwegian municipalities are responsible for safeguarding the mental health of their residents, but there are significant differences in the types of services they provide. Some municipalities are experiencing rapid growth, which may impact residents’ mental health and the accessibility of tailored mental health services. The aim of this study is to explore how a municipality’s rapid growth has affected residents’ mental health from both professionals’ and users’ perspectives.

**Methods:**

Focus group interviews were carried out in two groups with 14 participants from different professions and user organizations from one rapidly growing municipality. The focus group interviews were analyzed using qualitative content analysis.

**Results:**

The analysis resulted in three categories: Preparedness for complex life challenges, Importance of having a social network, and Changed threshold for access to health services.

**Conclusion:**

Health services are not sufficiently prepared to keep up with the complexity of challenges people are facing. No one is in charge, it is difficult to navigate the available services, and collaboration is not systematized. The threshold for getting mental health services has become higher.

## Introduction

Today’s rapid social development may have mental health consequences for individuals and groups in the population. The media reports stories about rural areas being depopulated, while city centers are becoming more urban and exposed to densification and centralization (1). Moreover, some municipalities in Norway are experiencing more growth than others, and this growth is expected to continue (2). In this study, the term “growing municipality” refers to a municipality that has experienced significant growth and transformation over the past two decades. It is characterized as a thriving community with a fast-paced development (3). The municipality in this study is characterized by busyness, socioeconomic pressure, unpredictability and unstable residential areas. In Norway, mental health services are public and organized together with general health services at two organizational levels, with separate financing. The municipalities are responsible for providing a range of health and care services to their residents. This includes primary healthcare services such as general practitioners, health clinics, and mental health services (4).

Rapid population growth in parts of the country has caused what might be called growing pains in several municipalities. These municipalities are experiencing financial pressure, which is partly due to a significant increase in the need for municipal health and care services (5). New and different needs have arisen, and more and more tasks are being transferred to the municipalities without this being fully financed (1, 6). Municipalities in Norway have a responsibility to safeguard the mental health of their residents. However, there are major differences between municipalities in terms of what kind of services they offer (5).

WHO defines mental health as “a state of wellbeing in which the individual realizes their own abilities, can cope with the normal stresses of life, can work and learn well and fruitfully, and contribute to their community” (7). Mental health conditions represent one of the most significant public health challenges in Europe and affect about 13% of the populations (8). Defining mental health problems is complex, and different terms such as mental disorder, mental disease, mental illness, and mental distress are used. Mental health problems affect thoughts, feelings and behaviors and interactions with others (9).

Various factors, both individual, social, and contextual, can affect residents’ mental health (9). Recent research suggests that serious mental health conditions may be linked to loneliness, social isolation, social support, the size of social networks and social capital at the individual level (10). Loneliness has been positively associated with depression (10) anxiety, self-harm, the risk of new episodes of depression, (11) suicide and dementia (12). Loneliness is described as a public health problem and several important predictors like age, gender, quality of social contact and socioeconomic status have been identified (11).

Social support has been identified as a crucial and decisive predictor of mental health and a key factor in preventing mental health problems (11, 13). The quality of social support appears to be of great importance for symptoms, recovery and social functioning (13). One study found that mental health services should develop services that are based more on social support (14).

Previous research highlights correlation between various socioeconomic, cultural and environmental factors and mental health in the population (15, 16). Key factors include social belonging and connection, experiencing social relationships as supportive and rewarding, trust in public institutions, perceived influence over authorities, satisfactory housing conditions, neighborhood noise, a sense of belonging to the area, housing satisfaction, and crime levels—all indicating a clear link between environmental characteristics and mental health (15). A previous study has shown that specific physical characteristics of the local environment, such as noise levels and access to green spaces, are important factors (16).

Mental health problems and disorders cause extensive personal suffering and predict a number of important outcomes, including physical disease, morbidity and mortality (17). A Norwegian report states that the prevalence of mental disorders is stable among adults. However, the results of the individual studies on which the report is based vary somewhat depending on region, age of participants and different weighting of the findings (9).

To explore and manage difficult feelings, individuals need a secure attachment figure. To become a subject, with their own world of experience, individuals depend on recognition from another subject (18). “All real living is meeting,” writes Buber. Moreover, Buber points out that individuals only become visible when they are with others (18). Individuals have a deep need to belong. In all stages of life, we are members of different groups, communities and social networks, such as families, workplaces, schools, and neighborhoods (19). Belonging to a community provides opportunities for engagement, which is important for mental health (20, 21). A previous review demonstrated how social relationships, group memberships and social identities have a beneficial impact by protecting mental health and enhancing psychological well-being (22). Previous research has found that social connectedness, social participation, and social interaction are important for adults’ mental health (23). According to the Norwegian Directorate of Health the municipalities should create open communities with a focus on acceptance and knowledge about mental health (24).

Social capital has become a cornerstone in community development (25). According to Robert D. Putnam, social capital refers to “connections among individuals – social networks and the norms of reciprocity and trustworthiness that arise from them” (26). Putnam and his followers consider social capital essential to building and maintaining democracy. The concept of social capital is divided into bonding and bridging capital. Bonding consists of strong communities characterized by trust, loyalty and boundaries. Bridging is about looser communities characterized by great diversity and which extend across some established boundaries and interests with varying degrees of trust, but which despite this can establish some common goals (26).

A high degree of social capital contributes generalized trust and reciprocity that affect both the individual and the societal level (26, 27). Social capital and health have been approached from different perspectives. Having more social capital can be associated with better health outcomes, predicts better mental and physical health, and is protective against mortality (28).

Rapid growth in municipalities can significantly impact residents’ mental health due to increased urbanization, changing social dynamics, and pressure on local resources. By focusing on how rapid growth influences mental health, this study seeks to provide insights that can guide municipalities in developing strategies to support the well-being of their residents. Understanding these dynamics is essential for creating resilient communities. Research on mental health among residents exists, but few studies have explored how the rapid growth of a municipality affects residents mental health from both professionals’ and users’ perspectives. Therefore, the aim of this study is to explore how the municipality’s rapid growth has affected residents’ mental health from both professionals’ and users’ perspectives.

## Methods

### Design

This study used an exploratory, descriptive design using qualitative methods. Qualitative methods are regarded as appropriate when the aim of the study is to understand an area that has been sparsely described and /or when the aim is to explore a phenomenon (29). This study aimed to gain insight into how professionals and representatives from user organizations experienced how the municipality’s rapid growth has affected the residents’ mental health. Qualitative methods were deemed suitable for capturing participants’ experience and perspective in depth. Data was collected using focus group interviews. Focus group interviews are a suitable method for gaining knowledge about people’s experiences, and how they think and feel about a specific issue (30). The focus group interviews were analyzed using qualitative content analyses as described by Graneheim and Lundmann (31). Qualitative content analyse is the analyses of the content of narrative data to identify prominent themes and patterns (32). Content analysis was chosen to systematically identify and interpret patterns and themes in the data. This method is well suited for exploring participants experiences and perspective and condense narratives into meaningful categories to address the research question (31).

### Setting

The municipality where the study was carried out is a society exposed to urbanization, rapid growth, and change. It is a local municipality that has experienced major upheavals and become an area of pressure (33). The municipality has a young population and high immigration. These are not primarily refugees or foreign immigrants, but people who voluntarily move to the municipality. In addition, this is a municipality with high population mobility. Many people move in and out (33), and the level of education among residents is low. The municipality has the highest unemployment rate in the region where it is located (33).

### Participants and procedure

A total of 14 participants including nine professionals (two nurses, two psychiatric nurses, two social workers, two social work leaders, one physician) and five representatives from different user organizations (two from mental health, three from the national association for careers in psychiatry) took part in two focus group interviews. Two of the professionals (one psychiatric nurses and one social worker) took part in both focus group interviews to ensure continuity. Each focus group consisted of eight participants. The expert and user perspectives were taken into account by including both professionals and user organizations in both focus group interviews.

All the participants were over 30 years old; the majority were over 50 years old with extensive work and life experience. Five were men and nine were women. This study focused on the adult population, but the participants represented and talked about different groups in the population, including children, young people, adults and older people. The participants distinguished between newcomers and those who had lived there for a long time. Involuntary or foreign immigrants were not discussed in this context.

### Data collection

Data were collected from February to June 2018. Both focus groups consisted of eight participants. In the literature, there is no consensus regarding the optimal numbers of focus group participants; it varies (34). The focus group interviews were held in a space in the municipality close to where the participants lived and worked.

Based on the research questions, an interview guide with a few open-ended questions was created, to allow participants to speak freely about the issue. Semi-structured interview is propriate to enable the development of new knowledge in an area where we have found little research (35). The interview guide is based on the researchers` experience, literature about mental health, and statistic about the specific municipality. Each question was designed to elicit rich, detailed discussions. According to Then et al., focus group interviews are often semi-structured interviews, so it was essential to create a guide to use in all focus groups interviews (36). The guide included three main questions (see Table 1). Follow-up questions were asked to obtain more detailed information. Examples of follow-up questions include: “Can you tell me more about that?” “Can you please explain?,” and so forth.

**Table 1:** Semi-structured interview guide.

No	Question
1	This municipality is undergoing rapid and comprehensive change and growth. How have you noticed this in your work?
2	What can be the possible mental health consequences for the resident in a rapid growing municipality?
3	The number of referrals to the municipality’s mental health services increased last year. What might be the reason for this increase in referrals?

In both focus group interviews the first author was the moderator and led the interview. One colleague participated in the interviews as an observer and helped to stay focused on the topic. The idea was to elicit a variety of information, as well as to bring out social interactions. In the interview situation, efforts were made to create an open and safe atmosphere and thereby facilitate the expression of personal and conflicting views in relation to the interview guide. The observer’s role was to help to maintain focus during the interview. She had some follow-up questions and after the interview the first author and the observer discussed the main impression of the interview. This was noted by the first author. Notes were also taken by the first author during the interviews on the topics that had been covered.

Both focus group interviews lasted about 1 h and were digitally recorded and transcribed verbatim by the first author.

### Data analysis

The analysis was performed by both authors. The interviews were analyzed using content analysis, as described by Graneheim & Lundmann (31, 38) which can vary in level of abstraction. Both authors participated in the analysis process and continuously discussed the process. First, the transcribed focus group interviews were read through several times by the authors to familiarize with the content, and to acquire an overall understanding and to ensure that they were transcribed accurately and correctly. The first author startet to analyse both interviews and then the second author was given these two interviews to analyze. Both authors identified and extracted meaning units which corresponded with the aim. These were words or sentences containing aspects that were related to each other through their context or content. The condensed meaning units were then abstracted and labeled with a code illustrating the key message embedded in the text. Both authors coded the interviews each and then came together and compared codes based on similarities and differences. We sorted the codes into categories and subcategories together. In an early phase we identified many sub-categories and categories that overlapped. These were discussed and revised and through this process, we agreed at the final set of categories. A category describes the content at a manifest level with a low degree of interpretation; however, the level of abstraction can vary (31). Although the analysis process was systematic, there was a back-and-forth movement between the whole and parts of the text. Quotations were used to support the descriptive categories.

### Methodological considerations

To describe trustworthiness in this study, the concepts such as credibility, dependability and transferability were used (31, 38).

Trustworthiness refers to the overall credibility and rigor of a study, ensuring that the research process and findings are presented in a way that convinces the reader of their reliability and validity. It is an overarching concept, based on a hermeneutic tradition, for describing whether the authors are able to present responsible results in such a way that they convince the reader (38). The researchers in this study have backgrounds as intensive care nurse and psychiatric nurse. Both authors have extensive experience in health education, and the first author also worked in the municipality’s mental health services a decade ago. While this background has enriched the study with valuable insights, it is possible that our preconceptions may have influenced the analysis, potentially leading to unintentional shortcuts. To strengthen trustworthiness, it has therefore been important to be systematic and to follow all the steps in the analysis process. During the analysis process we found that our proximity to the research phenomenon and insider knowledge, as described in the hermeneutic tradition, provided us with an enhanced ability to understand det meaning of individual statement through varying levels of interpretation (38). This can strengthen the credibility and authenticity of the study (38). However, the analysis in this study remained primarily descriptive and a deeper interpretive work represents an important next step for the field.

Credibility refers to how the investigation was carried out and confirmed (31). In this study there were only two focus groups, which can affect the results. Nevertheless, both focus group interviews provided substantial data. Although we cannot claim complete coverage, we believe that we have captured variation in the data. In our analysis, we observed that key categories were identified across both focus groups, with some variation in perspectives that enriched our understanding of the topic. While it is possible that additional focus groups could have uncovered new categories, we believe the data collected provides sufficient depth to address our research aims. The present study consisted of 14 participants, both female and male, representing different professions and users’ organizations. The professionals all have university level education and extensive work experience with mental health services and social work in the municipality. The participants from the users’ organizations had long experience within organization work and were familiar with users’ experiences. This will strengthen the credibility of the participants’ statement. Two participants participated in both focus group interviews, which can be seen as a strength. They could ensure continuity and that the same topic was covered. On the other hand, this could be seen as a limitation because they might have influenced the discussion. However, we found that they contributed to a fuller discussion. Both focus groups functioned well and provided rich data.

Dependability refers to the degree to which data change over time (37). The data in this study were collected from February 2018 to June 2018; this relatively short period may have helped ensure consistency in data collection. The participants were asked the same main questions, and follow-up questions were adapted to the discussions in the group. The data collected in 2018 may present a limitation, as society has undergone significant changes since then. The COVID 19 pandemic and its aftermath have had profound effects on individuals, the overall health situations, and the ways in which mental health problems are addressed. Our findings represent the context of rapid municipal growth and its impact on mental health services as it was prior to the COVID-19 pandemic. Since the data were collected in 2018, subsequent developments, including social distancing measures, rapid digitalization, and reorganization of mental health services, may have altered some of the challenges identified in this study. As such, the findings should be interpreted as a snapshot of pre-pandemic conditions, with limited applicability to the current post-pandemic landscape. However, the findings of this study remain highly relevant to today’s context, highlighting critical issues related to how rapid growth and urbanization can impact the mental health of a population. Although the data is from 2018, it is still relevant as the municipality in this study had the third largest population growth in Norway in 2024 (33).

Transferability refers to whether the results can be transferred to other settings or groups, which in this study means other municipalities. The current results came from different users’ organizations and different professionals from health and social services. In addition, the participants varied in terms of age, gender, and work experience, which can strengthen the transferability of the results. The question is how well these results represent other users’ organizations and health and social services in other rapidly growing municipalities in Norway. According to Statistics Norway (38), two out of three municipalities are expected to grow by 2050. Given that this study is based in a Norwegian context, its transferability should be evaluated with the understanding that contextual differences may exist between countries.

To appraise the quality of the study, the COREQ checklist was applied (39).

## Results

The analysis from the focus group interviews resulted in three categories related to the study aim: (1) Preparedness for complex life challenges; (2) Importance of having a social network; (3) Changed threshold for access to health services. The categories consisted of eight subcategories (see Table 2). The presentation will distinguish whether statements are from employees (emp) or from representatives from user/relative organizations (us/rel) and to which focus groups they belong (FG).

**Table 2:** Results with categories and subcategories.

Category	Preparedness for complex life challenges	Importance of having a social network	Changed threshold for access to health services
Sub-category	1) Life problems cause serious mental health challenges 2) The health services cannot follow the residents needs/complexity?	1) Being part of a community 2) Missing a neighbor who cares 3) Loneliness and isolation pose a risk	1) Tough priorities 2) Lack of collaboration, organization and knowledge of available services 3) The importance of easily available services.

### Preparedness for complex life challenges

The participants described how urbanization and rapid growth contributed to changes in residents’ lifestyles. These changes were linked to more complex life problems such as housing instability, financial stress and social isolation, which may affected mental health outcome. Many struggled with limited finances and social and psychological problems and needed help in relation to these challenges. In addition to mental health care, they needed services related to finances, education, work, and social life in general. One participant highlighted the interconnectedness of these factors stating: “*There are several external factors, such as housing, finances, small network etc… which people struggle with, and which are decisive for people’s health*” (FG1, emp). These factors interact in ways that reinforced residents’ struggles. For instance, rapid growth often leads to housing shortages or instability, which can further strain financial resources and limit opportunities for social connection. Participants also observed a correlation between rapid growth and the increasing complexity of municipal cases. One said: “*I see more often now that we get more complicated cases, which involve many services in the municipality. There are problems associated with life*” (FG1, emp). This suggests that while financial stress, housing instability and social isolation may be universal challenges for mental health services, their intensity and combination are shaped by the socioeconomic changes driven by rapid growth. The participants highlighted that an increasing number of residents were struggling with complex life problems for which no one was responsible. These challenges demanded more resources and necessitated collaboration with other professional actors to coordinate the work around residents’ needs.

The participants experienced that the complexity of the challenges residents faced contributed to increased violence, self-harm, drug addiction, and suicide among the population. They experienced an increase in urgent inquiries. One explained: “*The result is often that they resort to violence, often stabbing, against fellow residents or family, or they resort to self-harm*” (FG1, emp). The participants said they had experienced several suicides, which could be linked to long-term suffering that the health services had not been able to follow up. Another added: “*There is no one to help them*” (FGI, emp), reflecting a sense of systemic failure to address long-term suffering. This may suggest that the pressure associated with rapid growth creates an environment where unresolved challenges escalate into acute crises.

The participants experienced that the services provided by the municipality were not designed to address the challenges the residents struggled with. One participant expressed: “*There are several problems that the health services are not prepared for, such as lifestyle problems, isolation etc.*” (FG1, emp). Those with complex life challenges who needed help did not fit anywhere in the service offering. As another participant remarked, “*The services they need do not exist!*” (FG2, us/rel), underscoring the inadequacy of current offerings. Rapid growth appears to have outpaced the development of integrated services, leaving vulnerable populations without access to mental health services. The participant experienced that no one was in charge.

The participants highlighted a significant increase in residents’ need for services, which they linked to an escalating burden and pressure on municipal health service employees. This surge in demand was described as a longstanding trend, with one participant reflecting: “*Since the year 2000, I have really noticed, there are more people who need services; there is also a lot more pressure on everything you have to fix…. You must be able to get people out and back on their feet… there has been a lot more pressure, A LOT more!*” (FG2, emp). This statement underscores a dual challenge; not only is the volume increasing, but the complexity and urgency of these cases are also increasing. Since 2000 the population has doubled, which has had an impact on the mental health services. The municipality has been given more responsibility for services, while the economy has not kept pace with growth. The participants found it challenging to give residents what they needed. Furthermore, residents had more complex conditions that required closer collaboration between multiple services. The rapid growth had created confusion within the system, making it difficult to navigate the various services. Participants described the difficulty of providing the best possible treatment within the given framework, questioning which services should be provided. Additionally, they highlighted the importance of having the time and opportunity to contact each other to find solutions.

The employees experienced not being able to meet the complex needs of residents. One participant said: “*We need dialogue to find out about these complicated matters, because the individual service cannot do it alone*” (FG1, emp). Another explained: “*We do not have competence in everything. Then we need someone else to step in*” (FG2, emp). This statement reflects an important tension in the delivery of public services. The growing complexity of these cases demands a broader set of competencies, which no single professional or service can reasonably be expected to possess. This underscores the need for a systemic approach that integrates expertise across disciplines.

The participants described their municipality as a challenging place to live and to work. They noted the increasing pressure and challenges faced by residents and service providers, emphasizing that the demands are expected to grow even further.

### Importance of having a social network

The participants emphasized the importance of social networks for maintaining good mental health, highlighting the fundamental human need to be seen, valued and to belong. Having family, friends, work, and meaningful activities with others is essential for residents’ health, they pointed out. Networks were described as necessary to prevent and detect mental illness. One participant explained: “*It is important to be seen and to be part of a community, to mean something in a context is important for people’s mental health*” (FG2, us/rel). This statement captures the essence of belonging as an important determinant of mental health. The participants also pointed out gap in the current structures of health services, observing that residents often had unmet needs that extend beyond what the formal health care services can provide. The statement: “*People need someone who has time to come and talk to [them] in a slightly different way than we do in the health services*” (FG1, emp) highlight the importance of relational and non-clinical approaches to mental health support. This suggests a need for complementary systems that include peer support and volunteers who can offer time, empathy and connection in ways that the formal health services may not prioritize. Another participant said: “*Some say it bluntly: I have things I struggle with, and I want to participate in a community. Can I contribute something? Others who come here do not manage to tell them that they are struggling. They just say they want new friends*” (FG1, emp). This highlights the variability in how residents communicate their needs for social belonging. While some explicitly articulate their challenges and desire for involvement from the municipality, others wanted friendship to express their underlying struggles and need for belonging.

The importance of networks as a protective factor for mental health was strongly emphasized by the participants. This was reinforced by one participant who described how isolation can lead to illness: “*If you do not have a neighbor who cares or another point of contact who speaks up, then the possibility of becoming very ill increases*” (FG1, emp). This statement underscores the role of social networks not only in providing emotional support but also as a safety net. Another participant spoke out concerning people who are mentally ill: “*Not having much of a network is particularly challenging in relation to having someone to hold on to you when you have a mental illness*” (FG2, emp). Neighbors and other close contacts can act as an informal gatekeeper, detecting signs of mental illness and facilitating access to support or to health services. In this case, social networks are not just beneficial but vital for preventing severe mental health outcomes.

According to the participants, many residents lacked networks or had few close contacts in their daily lives. Several participants expressed concern about this development in the population. One described: “*Many who move here do not have roots here, or family, or friends, and [these connections have] to be rebuilt. It is demanding to build up a new network*” (FG1, emp). Several participants expressed concern that many people who moved to the municipality had no affiliations there. The absence of established social networks not only creates barriers to social belonging but may also intensify feelings of isolation and vulnerability, which are known risk factors for mental health illness.

Loneliness and isolation were identified by the participants as a growing concern across all age groups in the municipality, with important implications for mental health. Several participants described that people did not know each other, and many lacked a sense of belonging, had no one who cared, and suffered loneliness. This lack of belonging was described as a key factor to both the development and worsening of mental health illness without being discovered. They did not get the help they needed. As one participant said: “*The possibility of being seen is reduced in such a municipality. It is a result of isolation, the absence of neighbors who care, and an urban feel*” (FG1, emp). This suggests that the municipality may be experiencing a shift toward a more fragmented social structure where traditional community bonds are weakened, and social isolation becomes more normal.

The participants noted that certain groups of residents fell outside of the community in this municipality, lacking networks and the support needed to navigate life’s challenges. One described it like this: “*Many people feel lonely. It is an important signal! We see a lot of isolation*” (FG2, emp). This highlights the importance om recognizing loneliness, not just as an individual problem but as a social signal indicating broader systematic issues. Another participant emphasized the server consequences of such loneliness, stating: “*Many become very, very ill*” (FG2, emp).: The participants also pointed to the municipality’s structure and social challenges that worsened these issues. One explained: “*It is a municipality where it is easy to fall outside and many do*” (FG1, emp). This suggests that certain factors such as the municipality’s urban character or lack of social networks make it difficult for residents to find their place. The following statement highlights the challenges faced by residents of the municipality: “*You almost die before anyone notices you. Yes, you die before anyone hears you. If there is no one to pull you back up then…*” (FG2, us/rel). This powerful statement highlights the profound consequences of social isolation and the absence of support networks in rapidly changing municipality. Rapid growth often disrupts established social networks and creates a sense of disconnecting, especially for newcomers who may lack time or opportunities to build meaningful relationships.

The participants were clear that the services had an important role in networking as a measure to ensure the population’s mental health. They concluded that it was very important to have a network.

### Changed threshold for access to health services

Employees experienced the social developments they observed reflected a higher threshold for getting help. Employees described a shift from a low-threshold service, where residents could easily get help – to a high-threshold service requiring formal referrals, restrictive eligibility criteria, and limited durations of support. Until recently, they described, the health services had managed to keep up with the population’s increased needs. Nevertheless, they believed that the municipality had not been sufficiently prepared for how developments had led to an increased complexity in the population’s suffering. Many residents were refused services. In response to the rapidly growing population, several former low-threshold services in the municipality had been reorganized. One participant said: “*I have been working for 18 years now. There are radical changes from when we started as a service, where everyone could come to the door and get an offer*” (FG2, emp). The participants described tough priorities, which were set both in relation to who was offered services and for how long a time, with the result that many did not get the help they needed. This had resulted in a waiting list which they did not have before. Requiring a general practitioner (GP) referral, was described by one participant: “*We were a low threshold offer… Now there must be a referral from the GP…, to the allocation department…, … you get a decision that lasts so and so long*” (FG2, emp). This could introduce a barrier for residents seeking help. When the residents’ already struggling with mental health challenges, this extra step required to navigate this system may discourage them from seeking the help they need. One participant said it like this: “*We are a municipality with growing pains*” (FG2, emp).

Several participants described a mental health system that prioritized some groups over others, leaving some residents excluded. They felt the system did not take patients seriously and described it as being at the breaking point, unable to meet residents’ needs. One said: “*The health service was quite good, but then it got worse and worse, … became much more rigid…*” (FG2, us/rel). Another told us: “*The need is greater than the opportunity*” (FG2, us/rel). A third said: “*There will be more firefighting*” (FG2, emp). This reflects the employees’ recognition of the need to allocate limited resources strategically, ensuring that those with the most severe needs receive adequate care.

In addition, the participants worried about how the health services would be able to take care of people who had serious mental disorders. They clearly stated that these individuals had to be prioritized. One participant said: “*The most seriously mentally ill are also still there…. The sickest people live at home*” (FG1, emp). The participants who were employees of the health services were concerned about an increase in serious mental disorders and did not receive adequate help. Both the complexity and to get into position to help these patients are time consuming. This highlights the tension within the system, while prioritizing the most severe mental ill patients often comes at the expense of preventative care and support for less acute cases.

The participants shared that residents who needed mental health services did not know what services were available. In addition, they talked about how difficult it was to find alternative and complementary service offers because the employees of various services did not know each other. This lack of knowledge led to less adapted and flexible services and fewer residents got the help they needed. One participant said: “*I guess it was last summer that I struggled a lot. I called home nursing. They said, ‘No, I cannot do anything. You need to talk to someone who knows about mental health….’ Then I felt helpless*” (FG2, us/rel). This shows how fragmented communication and limited collaboration between services leave residents without the help they need, increasing frustration and feelings of helplessness. If services were not accessible and there was a lack of knowledge about available service options, it would significantly increase the barriers to seeking help. The participants found it challenging and difficult to make mental health services known, and this was a consequence of the rapid growth of the municipality.

The participants experienced that lack of organization and collaboration among various actors in the public, private, and nonprofit sectors of mental health services hindered accessibility and comprehensive treatment. They often struggled to know whom to contact, lacked time to contact other services, or were unaware of available resources. It was emphasized that effective collaboration is crucial as the people who have initial contact need to involve other services to ensure the best possible care. One participant said: “*It depended on collaboration, if people contact us when they find out about someone who could benefit from our service*” (FG1, emp). Another said: “*One must work to find the best in the individual service*” (FG2, us/rel). Findings from the analysis showed a municipality that had previously had some good low-threshold services, but several of them had been reorganized. While there were still some low-threshold services, there was a need for a more varied offer to capture the diversity of the residents.

Results from the focus group interviews were that low-threshold programs could help residents feel valued and provide them with meaningful activities. One participant referred to a low threshold offer which was partly user controlled. This was a place where you could meet others and participate in daily activities. It was about the importance of being seen, belonging to a community, experiencing handling various tasks, and contributing to society: “*If you can only bear to wipe the table after lunch that day, that’s what you do that day. And then that’s fine*” (FG2, us/rel). This statement reflects the importance of meeting people where they are and valuing small, achievable steps as a part of the recovery process. However, the participants also expressed concern about how rapid growth and increasing demand challenge the sustainability of such individualized approaches. Another participant added: “*It’s about having an open door where I can find a hand to hold. It’s about being seen…, as you do in close relationships. Without it, you will actually die before being heard. If there is no one there to pull you back up. Yes, those are my experiences*” (FG2, us/rel). These reflections emphasize the challenge of maintaining low-threshold person-centered services while facing the pressure of rapid growth and growing demand. They also illustrate how small social interactions, such as wiping the table and being seen, act as an important step toward building networks and belonging.

The participants described the necessity of low-threshold services to prevent mental disorders and to serve as a springboard to work and advancement in life. It was mentioned that prejudice and feelings of shame prevented people from seeking help with their mental health problems and it might therefore be easier to contact a low threshold offer.

## Discussion

The aim of this study has been to explore how a Norwegian municipality’s rapid growth has affected residents’ mental health from both professionals and users’ perspectives. The findings show that many participants perceived that society has become urbanized. This development had progressed quickly, and they felt that mental health services had not been adequately prepared for the complexity of the impact this change has had on the municipality’s residents. Additionally, it appears that many residents lacked social networks, which poses a risk for developing mental disorders. The rapid development of the municipality increased pressure on services, led to tough prioritization, and raised thresholds for access.

### Urbanization and “growing pains”—organization and collaboration

Participants lived in a municipality that had undergone rapid urbanization. They described a society that had transformed into a city-like community, with large new residential areas where residents did not know their neighbors. Louis Wirth describes urbanism as “a way of life” or as a social “lifestyle (40, 41). In an urban environment, a large number of people live in close proximity without knowing each other personally, leading to more transient interactions (41). Family connections are mostly outside the city (27). The urban lifestyle provides increased freedom but also results in little and looser contact between people. Networks are formed through work, school, and interests. This provides individual freedom, little social control (27), and the ultimate opportunity for growth and a comfortable, prosperous life (41). Those with good health, jobs, and economic stability benefit from such a society. If they are dissatisfied, they move, which creates more unstable residential areas (27). Established residents, who have lived in the municipality for a long time, may view the rapid development as a threat to the existing social order, which is rooted in equality and a sense of familiarity (42). Increased traffic, a rise in accidents and fatalities, as well as escalating violence, have contributed to growing concerns among residents (42).

This study can be linked to boomtown research, which shows that such rapidly growing municipalities are often associated with natural resource exploitation and are often rural. Since the 1970s, research has evolved from focusing on the economic benefits of development to looking at the harmful effects that the boom has had as a result of social change (42). Durkheim’s anomic society, marked by normlessness and lack of social cohesion (43) and Baumann’s liquid modernity, characterized by the constant movement of people, equipment, resources, and ideologies (44), are closely connected to such a society (42). Boomtown research refers to these challenges under the term “liquid dilemmas.” (44).

Louis Wirth’s description of urbanism as “a way of life,” Baumann’s liquid modernity and Durkheim’s anomic society are all draw parallels to the Norwegian welfare model. This model is based on values such as equal treatment, freedom of choice, self-determination, and transparency. Other important values include solidarity, legal certainty, and predictability. Central to the model are universal health and welfare schemes, equal opportunities for education and work, and economic security (1). Despite this foundations, municipalities are free to allocate resources within the various statutory services. This means that there are differences in the services received by residents in different municipalities (1). This is consistent with boomtown research, which shows that politicians in such communities often prioritize growth and a thriving local community, and emphasize cost-effective statutory services (3). However, such prioritization can lead to unmet needs and more complex problems.

According to the participants, lifestyle changes have led to residents experiencing new and more complex mental health disorders. It seems that the freedom of urban society poses a risk for those who find themselves outside the aforementioned arenas (27). Participants described residents struggling with challenges related to finances, housing, employment, lack of networks, and consequently social and mental functioning. This aligns with recent boomtown research, which indicates that stress and strain are disproportionately felt by individuals who are already economically disadvantaged (42). The urban lifestyle, characterized by high activity levels, fast pace, many stimuli, and high mobility did not seem to suit all residents (41, 42, 45). More and more areas of life that used to be taken care of by family and community have been outsourced to services that can be bought (e.g., psychologists). Family roles have been delegated to society and interacting with neighbors is no longer necessary. This shift can result in individuals withdrawing and experiencing isolation (45), which represent a significant risk to mental health (11–13). One study emphasizes that in such society, local municipalities must find new strategies to include their residents (3). These strategies must align with the speed required. The same study highlights barriers that hinder the necessary inclusion of welfare services in such a society. The participants in our study align with this study both pointing out to a significant gap between the needs of the residents and the capacity of services to meet those demands (3).

For some, urban development represents the ultimate opportunity for growth and a comfortable, prosperous life, while others perceive such a society as noisy, unpleasant, and filled with aggressive, unfriendly people and crime. Competition becomes tougher, and the bonds between residents weaken. People care less about each other. The gap between those who succeed and those who fail, as well as between the rich and the poor, widens (41, 46).

Participants observed an increase in mental disorders, violence, self-harm, substance abuse, and suicide, and linked this to the fact that many residents lacked coherence in their life and did not receive the healthcare services they needed. This is in line with research from Rocha et al. who found that the urban lifestyle causes distress among the population and reduces psychological well-being (47). The same study observed higher incidences of criminal activity; household problems, including domestic violence; alcohol abuse; and mental health issues. In this case, the healthcare system failed to meet residents’ needs and the health consequences of the agglomeration of people living in close quarters were apparently not fully understood (47). Our study indicates that mental health services faced a growing and complex set of challenges related to residents ‘changing lifestyle. Different lifestyles require different safety nets, meaning various forms of support (48). Participants experienced that healthcare services were not designed for the new set of challenges and were under significant pressure. Many residents needed assistance with various life challenges, but the mental health services lacked sufficient capacity and expertise in several of these domains. According to political guidelines, all citizens in Norway should have equal access to mental health services regardless of where in country they live (49). A previous study found that individuals diagnosed with a mental disorder or substance abuse had extensive care needs and required access to multiple different services (50). This highlights the importance of municipalities being able to offer services tailored to residents’ needs. Participants described a healthcare system on the brink, unable to meet residents’ needs.

Our finding aligns with previous research that emphasizes the importance of collaboration between services to address complex needs (51, 52). Participants in our study expressed concerns about the lack of collaboration between municipal services, which reflects finding from studies that have noted a challenges in interprofessional collaboration due to unclear roles and responsibilities (52, 53). These studies emphasize the systemic, organizational, and relational barriers that hinder effective collaboration, which are consistent with the experiences described by our participants. For example, participants in our study reported a desire for more collaboration but noted either a lack of time to initiate contact or insufficient awareness of what other services could offer. This reinforces the findings from previous studies that collaboration often depends on clear guidelines, well-structured organizational systems, and strong relationships between professionals (54).

Findings from the study suggest that improvements can be made by working on both organization and establishing regular meeting points between different services. This can contribute to more open communication, increased trust between stakeholders, and, most importantly, better awareness of available opportunities. As a result, more individually tailored and flexible services can be provided to residents (55).

### The importance of social capital factors

Based on Putnam’s theory, key elements such as trust, networks and norms are essential for ensuring residents’ social capital (26). It is interesting to examine how these elements are influenced in a municipality experiencing rapid growth and change in a municipality as well as how the development of bonding and bridging social capital is affected.

Many residents in the municipality studied lived alone and experienced loneliness. Social capital literature shows the importance of close and secure relationships as a buffer and an important resource in times of adversity (56). Such bonding capital, found in small and close network is particularly effective in helping individuals cope with and process difficult events, as well as in preventing mental illness (26, 56). Participants in our study described the positive association between being part of a community, feeling seen and valued in a meaningful context is associated with good mental health. However, they described that many residents lacked such bonding networks, opportunities for connection or neighbors who cared, leaving them without a sense of belonging.

Many residents suffered from loneliness, which could lead to development of mental health illness, and they did not receive the help they needed. Loneliness is reported to have negative impact on health and well-being (57). This is in line with previous research which confirms that loneliness and social isolation are associated with both increased and prolonged mental health problems across all age groups (58, 59). These findings highlight the importance of implementing measures to prevent and alleviate loneliness and social isolation.

A large proportion of the residents had few people in their social network. Research suggests that small size of one’s network can lead to a risk of lacking meaningful activities, which may cause loneliness and isolation (57). This can be linked to Putnam and his theories on bridging social capital and the importance of social networks. He argued that trust and reciprocity were crucial for a functioning society (26, 60). Putnam connected the determinants of trust, norms, and networks to the concept of social capital, later expanding it to include its impact on public health. However, research has been divided on this issue (60).

Recent studies suggest that the relationship between social capital and health depends on several factors, both cognitive and structural. The size of one’s social network and membership in institutions that bring people together and thus can function as bridging capital (26), appear to be essential factors for mental health. Mazumdar et al. found that variables such as resident homogeneity and geographically diverse social networks in a neighborhood may play an important role (61). Another study, however, showed that a neighborhood’s mixed socioeconomic status was negatively associated with depression and overall mental disorders, while urbanity was positively associated with depression and mental disorders (62). Some densely populated areas appear to attract a population with minimal local ties, who remain in the neighborhood only for a short period, primarily seeking easy access to various amenities (62). Mazumdar et al. use the term “outsider or stranger hypothesis” to describe this situation in such areas. It appears that ethnic diversity has a negative effect on social capital (61), most likely due to reduced trust and differing interpretations of societal norms.

Social capital factors appear to play a crucial preventive role in mental health when individuals face external stressors (60). We found that many residents had unmet needs that could have been addressed by entities other than healthcare services. These findings is in line with the research of Fiorillo et al., which shows that membership in an organization or community can promote mental well-being. Group participation enhances well-being by fostering acceptance, self-esteem, and security. Another study found that larger social networks also boost the likelihood of receiving emotional, informational and material support (63). Additionally, long-term participation in a community can help build lasting social bonds and enhance mental well-being (64). This suggests that community membership provides positive mental health benefits in both the short and long term.

## Conclusion

Findings from this study suggest that rapid growth and development may have consequences for residents’ mental health. In the study municipality, users and professionals reported increased challenges in terms of both what they experienced people struggling with and what opportunities the services have to meet their needs. They described a demanding society, where unstable residential areas, economic pressures, and changes in residents’ way of living created complex life challenges and mental health problems. They also experienced existing health services not sufficiently prepared to keep up with the complexity of the challenges people face. No one is in charge, it is difficult for users to find their way around the available services, and collaboration is not systematized. The threshold for accessing mental health services has become higher. Many residents are lonely, isolated and lack a sense of community. The novel findings in this study suggest that in a municipality experiencing rapid growth and urbanization, the speed of growth itself is the biggest challenge: Health services are not keeping pace with developments, residents’ needs, which are more complex than before, are not being adequately met, loneliness and isolation are increasing, and mental illness is given the opportunity to develop. Based on current theory and research, it seems likely that rapid growth and urban development in a municipality pose a risk to residents’ mental health.

This study has not explored residents’ experiences or examined all the factors that may affect residents’ mental health in a rapidly growing municipality. Further research in this field is therefore essential, particularly to understand how to address the complex set of challenges.

## Data Availability

The raw data supporting the conclusions of this article will be made available by the authors, without undue reservation.
